# Supplementary material for: Nonhomologous end-joining uses distinct mechanisms to repair each strand of a double strand break
Source: Nat Commun. 2025 Nov 24;16:11599. doi: 10.1038/s41467-025-66528-8 (PMC12748747; doi:10.1038/s41467-025-66528-8)
Supplement: Supplementary file 1 — Supplementary Infomation [file 41467_2025_66528_MOESM1_ESM.pdf]

Supplementary Materials for

Nonhomologous end-joining uses distinct mechanisms to repair each strand of a double strand break

Adam J Luthman *et al.*

Corresponding author: dale_ramsden@med.unc.edu

The PDF file includes:

Supplementary Figs. 1 to 4
Supplementary Tables 1 to 9

Supplementary Fig. 1. Validation of strand-specific NHEJ assay.

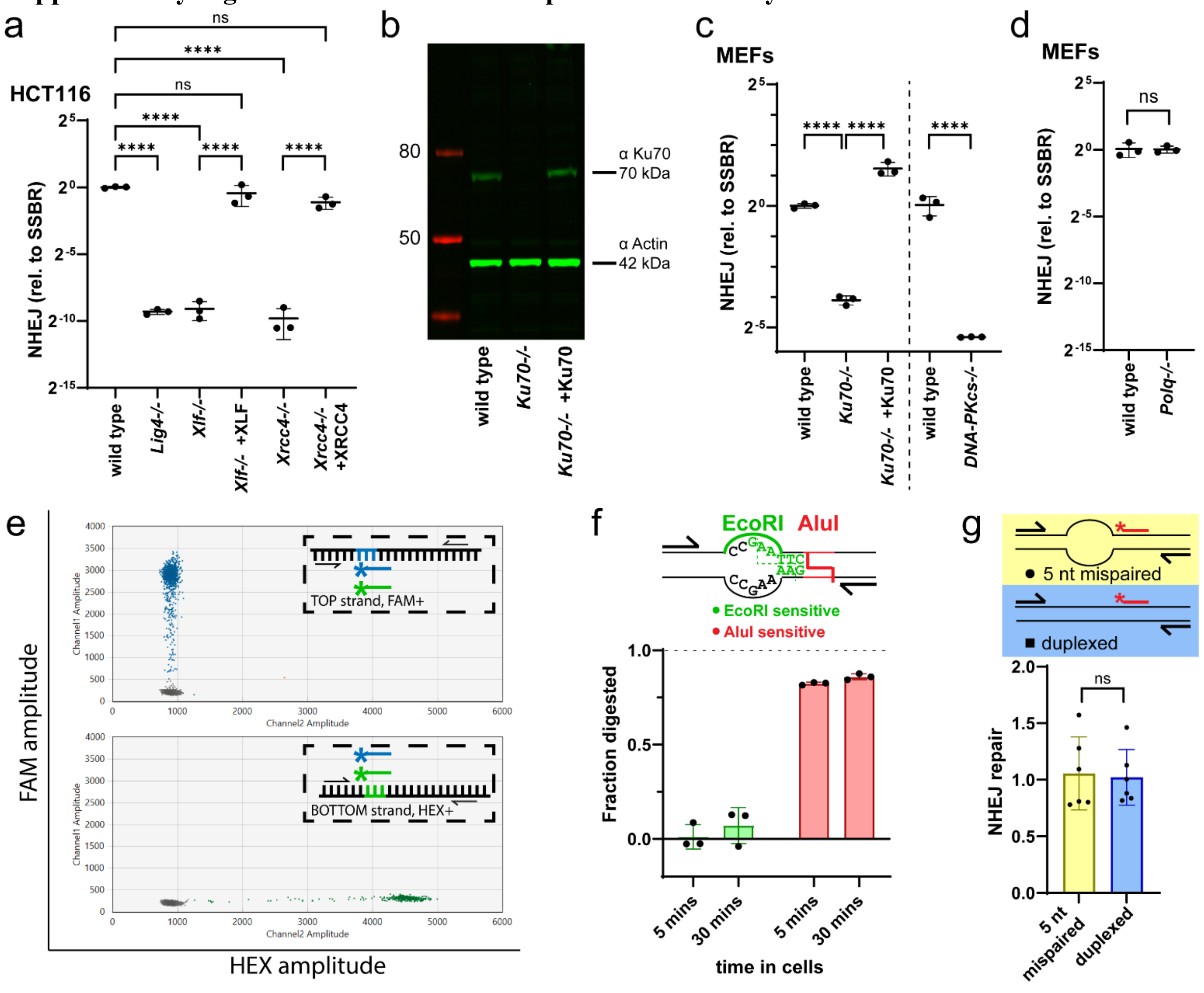

Supplementary Fig. 1. Validation of strand-specific NHEJ assay.

a. NHEJ substrates (3'CG/blunt) were introduced into human colorectal carcinoma cells (HCT116) that were wild type, individually deficient in LIG4, XLF, or XRCC4, or complemented by re-expression of XLF (+XLF) or XRCC4 (+XRCC4). Data represent the mean end joining efficiency ± s.d. from n=3 independent replicates, relative to wild type cells. Means were compared by one way ANOVA with Tukey's test for multiple comparisons. b. Western blot for knockout of Ku70 and subsequent complementation by lentiviral transduction. c. As in Supplementary Fig. 1a, except in parental (wild type) SV40 T-antigen transformed mouse embryo fibroblasts (MEFs), MEFs deficient in Ku70 or DNA-PKcs, or Ku70-deficient MEFs complemented by re-expression of Ku70. Means from n=3 independent biological replicates were compared by one way ANOVA with Dunnett's test for multiple comparisons (left of dashed line) or unpaired t-test (right of dashed line). Vertical dashed line separates independent experiments. d. As in Supplementary Fig. 1a except in SV40 T-antigen transformed MEFs proficient (wild type) or deficient in Pol θ (Polq-/-). Means from n=3 independent biological replicates were compared by unpaired t-test. e. Oligonucleotides consistent with repair by NHEJ of either the top strand (top panel, blue) or bottom strand (bottom panel, green) were amplified by digital droplet PCR in the presence of both the top strand-specific (FAM+) probe and the bottom strand-specific (HEX+) probe. f. An NHEJ substrate with a mispaired region for strand identification (see Fig. 1b) was introduced into wild type MEFs, then mock digested or digested with the noted restriction enzyme before

quantification of products by qPCR. Data are the mean fraction of restriction enzyme resistant NHEJ products (mock treated – restriction digested) ± s.d. from n=3 independent biological replicates. g. A substrate with a 5 nucleotide strand-ID mispaired region (yellow bars, see Fig. 1b) or a substrate with fully double-stranded DNA in this region (blue bars) was introduced into wild type MEFs. Data are plotted as mean total NHEJ ± s.d. of n=6 independent biological replicates. Means were compared by unpaired t-test. ****$p<0.0001$, ns = not significant for all panels. Source data for all relevant panels are provided as a Source Data file

Supplementary Fig. 2. Supplemental data for Figure 2 experiments.

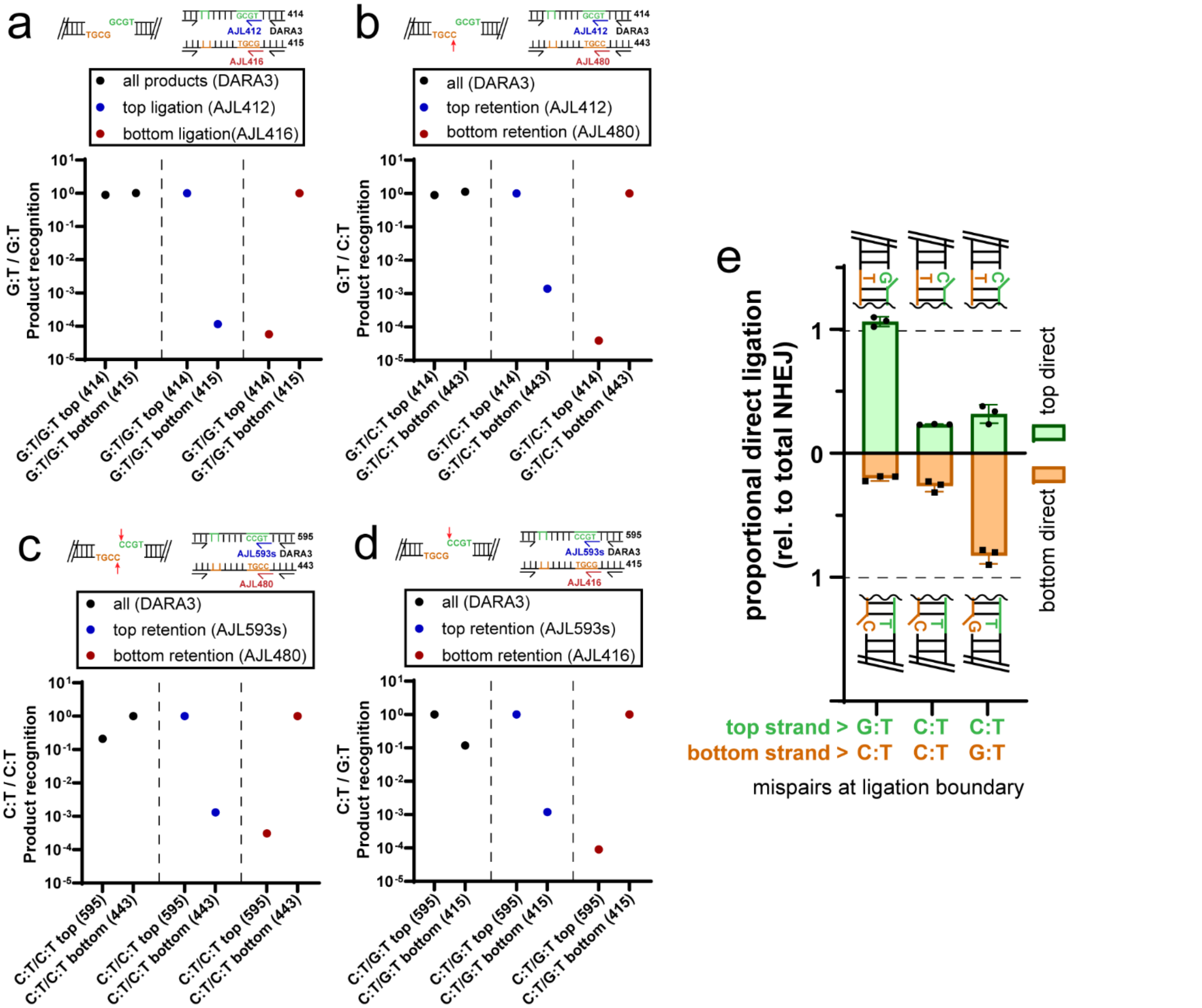

Supplementary Fig. 2. Supplemental data for Figure 2 experiments.

a. Control oligonucleotides identical to NHEJ products (model products) after top strand direct ligation of a 5’G:T mispair (414) or bottom strand direct ligation of a 5’G:T mispair (415), were amplified with a common forward primer and varying reverse primers, either one located upstream of the junction (amplifies all NHEJ products, DARA3), one specific to the top strand direct ligation junction (AJL412), or one specific to the bottom strand direct ligation product (AJL416). The data show the extent each strand’s product can be amplified by the other product-specific primer, relative to the extent of amplification observed using the cognate product specific primer. b. As in Supplementary Fig. 2a, except screening primers for a substrate with 5’G:T mispair for top strand ligation model product 414, recognized by primer AJL412) and 5’C:T mispair for bottom strand ligation (model product 443, recognized by primer AJL480). Altered nucleotides at the junction are indicated with a red arrow. c. As in Supplementary Fig. 2a, except screening primers for a substrate with 5’C:T mispair for top strand ligation model product 595, recognized by primer AJL593s) and 5’C:T mispair for bottom strand ligation (model product 443, recognized by primer AJL480). Altered nucleotides at the junction are indicated with a red arrow. d. As in Supplementary Fig. 2a, except screening primers for a substrate with 5’C:T mispair for top strand ligation model product 595, recognized by primer AJL593s) and 5’G:T mispair for bottom strand ligation (model product 415, recognized by primer AJL416). Altered

nucleotides at the junction are indicated with a red arrow. e. Direct ligation frequencies of NHEJ substrates with the indicated mispairs after 30 minutes in cells. Product frequency is represented as mean ± s.d. of n=3 independent biological replicates, relative to all repair products of the most abundant strand for each substrate as determined by digital PCR. Source data for all relevant panels are provided as a Source Data file.

Supplementary Fig. 3. Supplemental data for Figure 3 experiments.

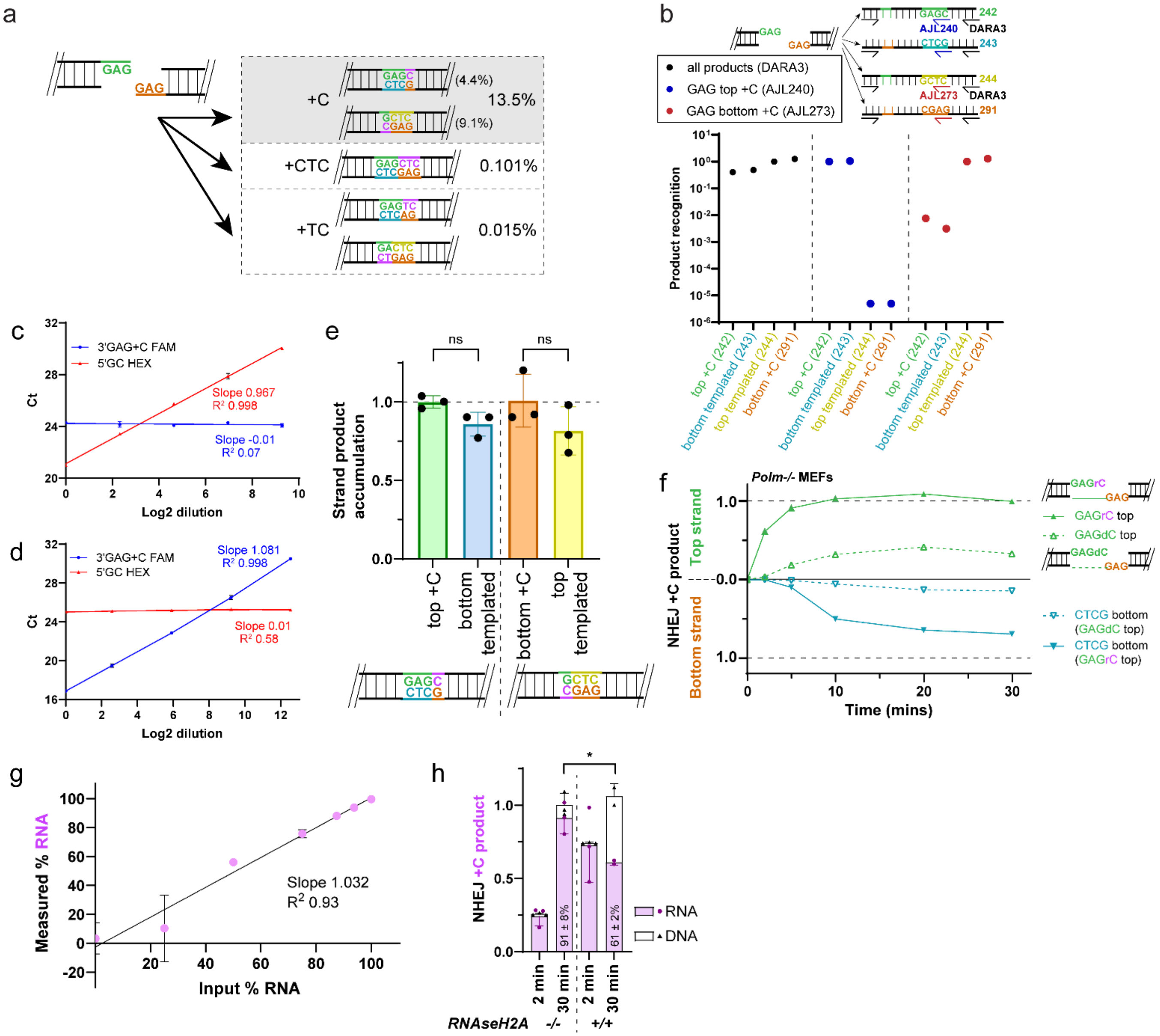

Supplementary Fig. 3. Supplemental data for Figure 3 experiments.

a. Next generation sequencing results from repair of 3'GAG/GAG substrate after 30 minutes in cells. The proportion of total repair for the four most abundant NHEJ products is shown as the mean of n=3 independent biological replicates. We additionally report the proportion of two products related to the Pol μ-dependent +C, but with longer complementary sequence additions (+TC and +CTC). b. As in Supplementary Fig. 2a, primers specific for the Pol μ-dependent +C product on each strand (top strand: AJL240, bottom strand AJL273) or a primer to amplify all NHEJ junctions (DARA3) were screened by amplification of control oligos which model NHEJ products for the Pol μ-dependent top strand +C (AJL242) and a bottom strand complementary to this sequence (AJL243), or the reciprocal products: a bottom strand Pol μ-dependent +C (AJL244) and a top strand complementary to this sequence (AJL291). c. Standard curve validating linearly responsive multiplexed qPCR reactions of serial dilutions of 5'GC direct ligation model product oligo titrated into a constant amount 3'GAG+C model product oligo. GAG+C product amplified with AJL240 for top strand +C product. Data are mean ± s.d of n=3 independent technical replicates. Data were analyzed by simple linear regression. d. As in panel c, except titrating

3’GAG+C model product oligo into a constant amount of 5’GC direct ligation model product oligo. GAG+C product amplified with AJL240 for top strand +C product. Data are mean ± s.d of n=3 independent technical replicates. e. Abundance of top strand +C repair (green) and bottom strand semi-conservative repair (blue) or the reciprocal products (orange and yellow, respectively, see Fig. 3a) of 3’GAG/GAG substrate measured by digital PCR after 30 minutes in cells. Data are normalized to the mean abundance of top strand and plotted as mean ± s.d. of n=3 independent biological replicates. Means were compared by one way ANOVA with Tukey’s test for multiple comparisons. Vertical dashed line separates independent experiments. f. Relative strand-specific repair plotted as in Fig. 3d for 3’ non-complementary overhangs (3’GAG/GAG). One strand break terminus possessed an added 3’-ribonuceleotide C (3’GAGrC / GAG, solid lines) or a deoxynucleotide C (3’GAGdC / GAG, dashed lines) to simulate Pol μ activity on this substrate upon electroporation into *Polm*$^{-/-}$ MEFs. For either top (green, above x-axis) or bottom strand (blue, below x-axis), abundance of each strand is expressed relative to the top strand Pol μ-dependent product after 30 minutes in cells, as determined by digital PCR. g. Standard curve for detection of embedded RNA in NHEJ products. Fully DNA oligos or similar oligos containing 1 nt of embedded RNA were mixed in ratios as indicated. Data are mean ± s.d of n=3 independent technical replicates. Data were analyzed by simple linear regression. h. RNA content for Polμ-dependent top strand +C product on 3’GAG/GAG substrate after 2 and 30 minutes in RNaseH2A proficient and deficient MEFs, as determined by alkali digestion of embedded ribonucleotides. Data are mean ± s.d of n=3 independent biological replicates, except for RNA content in *RNaseH2A*$^{+/+}$ cells (far right), which contains 2 biological replicates. *p=0.034, ****p<0.0001, ns=not significant for all panels. Vertical dashed line separates independent experiments. Source data for all relevant panels are provided as a Source Data file.

Supplementary Fig. 4. Supplemental data for Figure 4 experiments.

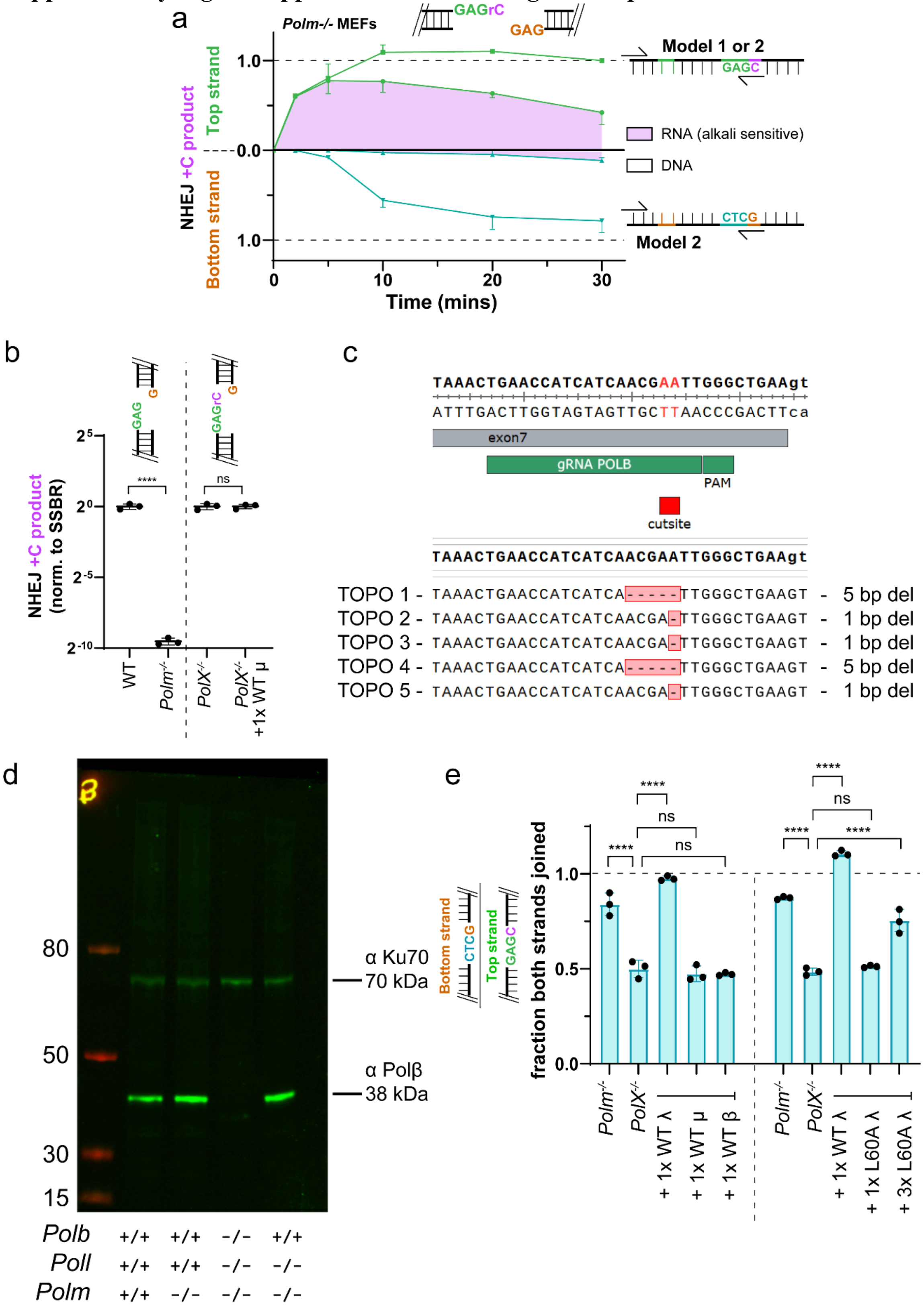

Supplementary Fig. 4. Supplemental data for Figure 4 experiments.

a. Relative strand-specific repair plotted as in Fig. 3d for 3' non-complementary overhangs (3'GAG/GAG and derivatives thereof). One strand break terminus possessed an added 3'-ribonucleotide C (3'GAGrC/GAG) to simulate Pol μ activity on this substrate upon electroporation into Polm-/- MEFs. For either top (green, above x-axis) or bottom strand (blue, below x-axis), RNA-containing fraction is shaded purple, while DNA fraction is left unshaded. Abundance of each strand is expressed relative to the total (mock treated, purely DNA and RNA-embedded products both) top strand Pol μ-dependent product after 30 minutes in cells, as determined by digital PCR. b. NHEJ +C product abundance in the presence or absence of Pol μ following introduction of the substrate represented in Fig. 4a (3'GAG/G, right) and used in Fig. 4b, or a variant of this substrate terminated in a 3'riboC. Data show mean ± s.d of n=3 independent biological replicates and were compared by unpaired t-test for experiment independently (each side of the dashed line). Vertical dashed line separates independent experiments. c. Polb allele sequences in PolX-/- (Polb-/- Poll-/- Polm-/-) MEFs following introduction of Cas9-sgRNA targeting exon 7 of Polb, displaying frameshift mutations of 1 bp or 5 bp deletions. d. Western blot for Pol β in wild type MEFs, Polm-/- MEFs, PolX-/- MEFs, and Poll-/- Polm-/- MEFs; Ku70 as loading control. e. As in Fig. 4b, except after 30 minutes in cells. Data are plotted as the mean fraction of repair products with both strands joined ± s.d. from n=3 independent biological replicates. Means were compared by one way ANOVA with Dunnett's test for multiple comparisons. ****p<0.0001, ns = not significant for all panels. Source data for all relevant panels are provided as a Source Data file.

Supplementary Table 1. sgRNA and genomic screening oligos.

Oligos	Name	Sequence (5’ → 3’)
sgRNA-*Polb*	AJL422	/AltR1/rUrGrArArCrCrArUrCrArUrCrArArCrGrArArU rUrGrUrUrUrUrArGrArGrCrUrArUrGrCrU/AltR2/
Polb sequencing oligo (fwd)	AJL423	ACTGAACAGGGAAGGTAACAAA
Polb sequencing oligo (rev)	AJL424	GAGAGGCCATGTATCTGAAAGAG
sgRNA-*Prkdc* (DNA-PKcs)	AJL327	/AltR1/rArCrUrArArGrArArGrUrArCrCrArGrArCrUrC rArGrUrUrUrUrArGrArGrCrUrArUrGrCrU/AltR2/
sgRNA-*XRCC6* (Ku70)	KKC277	/AltR1/rCrArArCrUrUrCrArCrCrArCrArGrGrCrUrArA rCrGrUrUrUrUrArGrArGrCrUrArUrGrCrU/AltR2/
	AJL418	/AltR1/rArCrGrGrArUrArCrArUrCrArArArGrCrCrUrC rCrGrUrUrUrUrArGrArGrCrUrArUrGrCrU/AltR2/

Supplementary Table 2.

Five most frequent top strand NHEJ products for the substrate used in Fig 2a-d with head and tail 5’GCGT overhangs. Junction sequences represented as flanking tail DNA sequence, retained overhang sequence between colons, followed by flanking head sequence. The average frequency (Freq., %) of each sequence and standard deviation (s.d.) are from three independent experiments. Mechanism column defines NHEJ products as Direct, joining of 5’G:T mispair in top strand as in Fig 2a; Direct (templated), joining of 5’G:T mispair in bottom strand which is then used as template for top strand product; 5’ editing, replacement of terminal 5’G with complementary A in top strand. Deleted sequence noted by dashes. Alignment + fill in describes a. product joined after alignment of 2 terminal head and tail overhang nucleotides followed by fill-in and ligation.

Substrate: 5’GCGT/5’GCGT (Fig. 2a-d)			
Junction	Freq.(%)	s.d.(%)	Mechanism
AGAGCTGAGGCAT:GCGT:TAGCTTAGCTGTA	96	1.8	Direct
AGAGCTGAGGCAT:ACGC:TAGCTTAGCTGTA	2.4	1.1	Direct (templated)
AGAGCTGAGGCAT:ACGT:TAGCTTAGCTGTA	0.39	0.42	5’ editing
AGAGCTGAG----:GCGT:TAGCTTAGCTGTA	0.34	0.14	Deletion
AGAGCTGAGGCAT:ACGCGT:TAGCTTAGCTGTA	0.31	0.12	Alignment + fill-in

Supplementary Table 3. NGS oligo sequences.

**NGS ligation adapter**	**Name**	**Sequence (5' → 3')**
short oligo	AJL528	/5phos/GCGGACTGTCCAAGTTTCAGATTGCGTA-3'
long oligo, with UMI (N_{10})	AJL529	5'CTTCGGTGACGTTGTCGAGNNNNNNNNNNTACGCAATCTGAAACTTGGACAGTCCGCTGC-3'
**Sequencing primers**	**Name**	**Sequence (5' → 3')** [a]
P5/i5, **TATCCT index +0 phase**	AJL518	5'AATGATACGGCGACCACCGAGATCTACACtctttccctacacgacgctcttccgatct**TATCCT**AACTTCGTGAGGACACACT-3'
P5/i5, **CTAAGC index +1 phase**	AJL519	5'AATGATACGGCGACCACCGAGATCTACACtctttccctacacgacgctcttccgatct**ACTAAGc**AACTTCGTGAGGACACACT-3'
P5/i5, **GCGTAA index +2 phase**	AJL520	5'AATGATACGGCGACCACCGAGATCTACACtctttccctacacgacgctcttccgatct**GAGCGTaa**AACTTCGTGAGGACACACT-3'
P5/i5, **TTATGC index +3 phase**	AJL521	5'AATGATACGGCGACCACCGAGATCTACACtctttccctacacgacgctcttccgatct**GCTTTAtgc**AACTTCGTGAGGACACACT-3'
P5/i5, **GAGCCT index +4 phase**	AJL522	5'AATGATACGGCGACCACCGAGATCTACACtctttccctacacgacgctcttccgatct**AGCTCTatta**AACTTCGTGAGGACACACT-3'
P7/i7, **TCGCCT index +0 phase**	AJL523	CAAGCAGAAGACGGCATACGAGATgtgactggagttcagacgtgtgctcttccgatct**TCGCCT**CTTCGGTGACGTTGTCGAG-3'
P7/i7, **ACTAAG index +1 phase**	AJL524	5'CAAGCAGAAGACGGCATACGAGATgtgactggagttcagacgtgtgctcttccgatct**ACTAGTa**CTTCGGTGACGTTGTCGAG-3'
P7/i7, **TAGCTC index +2 phase**	AJL525	5'CAAGCAGAAGACGGCATACGAGATgtgactggagttcagacgtgtgctcttccgatct**TAGCTCag**CTTCGGTGACGTTGTCGAG-3'
P7/i7, **CTAAGG index +3 phase**	AJL526	5'CAAGCAGAAGACGGCATACGAGATgtgactggagttcagacgtgtgctcttccgatct**CTAAGGagt**CTTCGGTGACGTTGTCGAG-3'
P7/i7, **GCTATA index +4 phase**	AJL527	CAAGCAGAAGACGGCATACGAGATgtgactggagttcagacgtgtgctcttccgatct**GCTATAgcga**CTTCGGTGACGTTGTCGAG-3'
P5 Enrichment primer	-	5'-AATGATACGGCGACCACCGA-3'
P7 Enrichment primer	-	5'-CAAGCAGAAGACGGCATACGAGAT-3'

[a] Barcodes indicated in **BOLD**, phasing nucleotides indicated in **lowercase bold**.

Supplementary Table 4. NGS library primers and barcodes used for de-mulitplexing.

Substrate	Time	Fwd primer	Fwd barcode [a]	Rev primer	Rev barcode [a]
5'G:T-G:T	2 minutes	AJL519	ACTAAGcAAC	AJL526	GactCCTTAG
		AJL519	ACTAAGcAAC	AJL527	tcgcTATAGC
		AJL519	ACTAAGcAAC	AJL525	AGctGAGCTA
	-	-	-	-	-
	30 minutes	AJL521	GCTTTAtgcA	AJL523	GAAGAGGCGA
		AJL521	GCTTTAtgcA	AJL524	AAGtACTAGT
		AJL521	GCTTTAtgcA	AJL525	AGctGAGCTA
**Substrate**	**Time**	**Fwd primer**	**Fwd barcode [a]**	**Rev primer**	**Rev barcode [a]**
5'G:T-C:T	2 minutes	AJL521	GCTTTAtgcA	AJL526	GactCCTTAG
		AJL521	GCTTTAtgcA	AJL527	tcgcTATAGC
		AJL522	AGCTCTatta	AJL523	GAAGAGGCGA
	-	-	-	-	-
	30 minutes	AJL522	AGCTCTatta	AJL524	AAGtACTAGT
		AJL522	AGCTCTatta	AJL526	GactCCTTAG
		AJL522	AGCTCTatta	AJL527	tcgcTATAGC

[a] Phasing nts shown in lowercase.

Supplementary Table 5. NGS analysis metrics.

Substrate	Time	Input molecules [a]	Reads	UMIs passing 2% threshold [b]	% 1 stranded [c]	% 2 stranded [d]	% 3+ stranded [e]
5'G:T-G:T	2 minutes	5000	12948931	5411	29.99	69.54	0.46
		5000	14990014	5257	27.18	72.47	0.34
		5000	17071326	5107	31.37	68.34	0.29
	-	-	-	-	-	-	-
	30 minutes	5000	15768422	5487	50.36	46.93	2.72
		5000	18012032	5341	32.88	64.95	2.17
		5000	17406702	5395	34.51	63.99	1.50
**Substrate**	**Time**	**Input molecules [a]**	**Reads**	**UMIs passing 2% threshold [b]**	**% 1 stranded [c]**	**% 2 stranded [d]**	**% 3+ stranded [e]**
5'G:T-C:T	2 minutes	5000	19527613	2934	73.89	26.04	0.07
		5000	14314164	2234	76.32	23.63	0.04
		5000	14901634	3471	76.75	23.22	0.03
	-	-	-	-	-	-	-
	30 minutes	5000	16231307	5793	51.29	48.49	0.22
		5000	13249211	5677	53.41	46.38	0.21
		5000	12158941	5354	60.53	39.15	0.32

[a] Measured by digital PCR (see methods)
[b] 2% oversampling threshold determined by input molecules / read counts
[c] Single strand-repaired intermediates (% of all UMIs analyzed)
[d] Both strands repaired, duplexed (% of all UMIs analyzed)
[e] Erroneous product classification, mutations occurring in UMI during sample processing (% of all UMIs analyzed)

Supplementary Table 6. Duplex NGS analysis displaying frequency of indicated repair products.

Substrate	Time	sample	overhang retention	both direct [a]	top direct only [b]	bottom direct only [b]	top direct + bottom templated [c]	bottom direct + top templated [c]
5'G:T-G:T	2 minutes	rep1	0.9701	0.7135	0.1975	0.0845	0.0043	0.0002
		rep2	0.9755	0.7349	0.2011	0.0580	0.0053	0.0006
		rep3	0.9740	0.6940	0.2070	0.0901	0.0084	0.0004
		**avg**	**0.9732**	**0.7142**	**0.2019**	**0.0776**	**0.0060**	**0.0004**
	**-**	**-**	**-**	**-**	**-**	**-**	**-**	**-**
	30 minutes	rep1	0.9140	0.5082	0.3095	0.1551	0.0238	0.0035
		rep2	0.9335	0.6540	0.2535	0.0536	0.0340	0.0048
		rep3	0.9462	0.6536	0.2654	0.0556	0.0228	0.0026
		**avg**	**0.9313**	**0.6052**	**0.2761**	**0.0881**	**0.0269**	**0.0036**
**Substrate**	**Time**	**sample**	**overhang retention**	**both direct [a]**	**top direct only [b]**	**bottom direct only [b]**	**top direct + bottom templated [c]**	**bottom direct + top templated [c]**
5'G:T-C:T	2 minutes	rep1	0.9584	0.2229	0.6967	0.0464	0.0340	0.0000
		rep2	0.9624	0.2111	0.7306	0.0370	0.0213	0.0000
		rep3	0.9588	0.2018	0.7227	0.0534	0.0221	0.0000
		**avg**	**0.9599**	**0.2119**	**0.7167**	**0.0456**	**0.0258**	**0.0000**
	**-**	**-**	**-**	**-**	**-**	**-**	**-**	**-**
	30 minutes	rep1	0.9285	0.0241	0.4844	0.0119	0.4783	0.0014
		rep2	0.9354	0.0170	0.4921	0.0154	0.4739	0.0016
		rep3	0.9229	0.0309	0.5719	0.0196	0.3757	0.0019
		**avg**	**0.9289**	**0.0240**	**0.5161**	**0.0156**	**0.4426**	**0.0016**

[a] Both direct = model 1
[b] Top/bottom direct only = single strand-repaired intermediate
[c] Direct + templated = model 2

Supplementary Table 7.

Five most frequent top strand NHEJ products for the substrate used in Fig. 2e-f with head 5'GCGT overhang and tail 5'CCGT overhang. Junction sequences, frequencies, and mechanisms as in Supplementary Table 2.

Substrate: 5'GCGT/5'CCGT (Fig. 2e-f)			
Junction	Freq.(%)	s.d.(%)	Mechanism
AGAGCTGAGGCAT:GCGT:TAGCTTAGCTGTA	96	0.34	Direct
AGAGCTGAGGCAT:ACGG:TAGCTTAGCTGTA	0.73	0.22	Direct (templated)
AGAGCTGAGGCAT:ACGT:TAGCTTAGCTGTA	0.67	0.11	5' editing
AGAGCTGAG----:GCGT:TAGCTTAGCTGTA	0.64	0.07	Deletion
AGAGCTGA----------------AGCTGTA	0.22	0.06	Deletion

Supplementary Table 8.

Five most frequent top strand NHEJ products for the substrate used in Fig. 3a-d, with head and tail 3'GAG overhangs. Junction sequences, frequencies, and mechanisms as in Supplementary Table 2.

Substrate: 3'GAG/3'GAG (Fig. 3a-d, Supp. Fig. 3a-g)			
Junction	Freq.(%)	s.d.(%)	Mechanism
AGAGCTGAGGTC--------CACCTTAGCTGT	25	3.5	Deletion
AGAGCTGAGGTC:GCTC:TCCACCTTAGCTGT	10	1.1	+C (templated)
AGAGCTGAGGTC------TCCACCTTAGCTGT	6.0	1.4	Deletion
AGAGCTGAG-----------------AGCTGT	6.0	0.92	Deletion
AGAGCTGAGGTC:GAGC:TCCACCTTAGCTGT	5.6	0.72	+C

Supplementary Table 9. Data collection and refinement statistics.

**Pol λ + 1nt SSB** [a]	
PDB ID code	9NPU
**Data collection**	
Space group	$P2_12_12_1$
Unit cell dimensions	-
a, b, c (Å)	56.20, 59.92, 140.10
α, β, γ (˚)	90, 90, 90
Resolution range [b] (Å)	41.01-1.63 (1.69-1.63)
R_{merge} [b]	0.11 (154.1)
R_{pim} [b]	3.5 (52.6)
$CC_{1/2}$ [b]	0.99 (0.407)
Mean I/σI [b]	11 (1.4)
Completeness [b] (%)	100 (100)
Redundancy [b]	10.4 (9.1)
**Refinement**	
Resolution [b] (Å)	40.99-1.63
No. reflections	60225
R_{work}/R_{free} (%)	17.81/19.92
No. atoms	-
Protein	4948
DNA	696
Incoming nucleotide	37
Ions	4
Water	267
B-factors ($Å^2$)	-
Protein	35.95
DNA	26.13
Incoming nucleotide	17.86
Ions	30.10
Water	33.06
RMS deviations	-
Bond lengths (Å)	0.012
Bond angles (˚)	1.083
Ramachandran plot	-
Most favorable (%)	99.07
Allowed (%)	100

[a] A single crystal was used for data collection.
[b] Values in parenthesis are for the last resolution shell.
