# Supplementary material for: Nonhomologous end-joining uses distinct mechanisms to repair each strand of a double strand break
Source: Nat Commun. 2025 Nov 24;16:11599. doi: 10.1038/s41467-025-66528-8 (PMC12748747; doi:10.1038/s41467-025-66528-8)
Supplement: Supplementary file 2 — Description of Additional Supplementary Files [file 41467_2025_66528_MOESM2_ESM.docx]

Supplementary Movie 1.

Animation of parallel, symmetric repair by NHEJ (model 1; depicted in Fig. 4, left).

Supplementary Movie 2.

Animation of ordered, semi-conservative repair by NHEJ (model 2; depicted in Fig. 4, right).

**Supplemental Dataset 1.**

Description of oligonucleotides used with sequences, modifications, and key features noted. 5’Phos, 5’ phosphate. 56-FAM, 5’ 6-fluorescein. HEX, Hexafluorescein.
