# Supplementary material for: Nonhomologous end-joining uses distinct mechanisms to repair each strand of a double strand break
Source: Nat Commun. 2025 Nov 24;16:11599. doi: 10.1038/s41467-025-66528-8 (PMC12748747; doi:10.1038/s41467-025-66528-8)
Supplement: Supplementary file 6 — Reporting Summary [file 41467_2025_66528_MOESM6_ESM.pdf]

nature portfolio

Corresponding author(s): Dale A. Ramsden

Last updated by author(s): Jul 8, 2025

Reporting Summary

Nature Portfolio wishes to improve the reproducibility of the work that we publish. This form provides structure for consistency and transparency in reporting. For further information on Nature Portfolio policies, see our Editorial Policies and the Editorial Policy Checklist.

Statistics

For all statistical analyses, confirm that the following items are present in the figure legend, table legend, main text, or Methods section.

n/a	Confirmed	
☐	☒	The exact sample size (*n*) for each experimental group/condition, given as a discrete number and unit of measurement
☐	☒	A statement on whether measurements were taken from distinct samples or whether the same sample was measured repeatedly
☐	☒	The statistical test(s) used AND whether they are one- or two-sided<br>*Only common tests should be described solely by name; describe more complex techniques in the Methods section.*
☒	☐	A description of all covariates tested
☐	☒	A description of any assumptions or corrections, such as tests of normality and adjustment for multiple comparisons
☐	☒	A full description of the statistical parameters including central tendency (e.g. means) or other basic estimates (e.g. regression coefficient) AND variation (e.g. standard deviation) or associated estimates of uncertainty (e.g. confidence intervals)
☐	☒	For null hypothesis testing, the test statistic (e.g. *F*, *t*, *r*) with confidence intervals, effect sizes, degrees of freedom and *P* value noted<br>*Give P values as exact values whenever suitable.*
☒	☐	For Bayesian analysis, information on the choice of priors and Markov chain Monte Carlo settings
☒	☐	For hierarchical and complex designs, identification of the appropriate level for tests and full reporting of outcomes
☒	☐	Estimates of effect sizes (e.g. Cohen's *d*, Pearson's *r*), indicating how they were calculated

Our web collection on statistics for biologists contains articles on many of the points above.

Software and code

Policy information about availability of computer code

Data collection	QuantStudio RealTime PCR Software (Applied Biosystems, v1.7.2), QX Manager Software Standard Edition (BioRad, v1.2 and v2.2), ImageStudio (Licor Biosciences, v5.2), AlphaFold Multimer (COSMIC2)
Data analysis	Excel (Microsoft), Prism (GraphPad, v10.5.0), QuantStudio RealTime PCR Software (Applied Biosystems, v1.7.2), QX Manager Software Standard Edition (BioRad, v1.2 and v2.2), ImageJ (v1.54g) with Java (v1.8.0), PyMOL (Schrödinger, LLC, v3.1.1), autoPROC toolbox (Global Phasing Limited), Phaser (University of Camrbidge, v2.7.17), COOT (GNU GPL, Paul Emsley), Phenix (v1.21), Web 3DNA (v2.0)<br><br>Initial processing of high throughput sequencing data was performed with CLC Genomics Workbench (Qiagen, version 12.0). Custom Python code was developed using PyCharm Community Edition (JetBrains, 2021.2.0.1) and is available at permanent repository Zenodo (DOI: 10.5281/zenodo.15528810) and at community repository on Github (https://github.com/aluthman/Ramsden-Lab.git).

For manuscripts utilizing custom algorithms or software that are central to the research but not yet described in published literature, software must be made available to editors and reviewers. We strongly encourage code deposition in a community repository (e.g. GitHub). See the Nature Portfolio guidelines for submitting code & software for further information.

Data

Policy information about availability of data

All manuscripts must include a data availability statement. This statement should provide the following information, where applicable:

- Accession codes, unique identifiers, or web links for publicly available datasets
- A description of any restrictions on data availability
- For clinical datasets or third party data, please ensure that the statement adheres to our policy

Data availability: Raw data generated in this study for duplex sequencing experiments have been deposited in NCBI Sequence Read Archive under accession code PRJNA1268600 (https://www.ncbi.nlm.nih.gov/bioproject/PRJNA1268600). Coordinates and structure factors have been deposited into the Protein Data Bank under PDB ID code 9NPU (hyperlink). Source data are provided with this paper. All other materials are available upon request.

Code availability: Analysis code is available at (https://github.com/aluthman/Ramsden-Lab.git) and at permanent repository Zenodo (https://doi.org/10.5281/zenodo.15528811).

Research involving human participants, their data, or biological material

Policy information about studies with human participants or human data. See also policy information about sex, gender (identity/presentation), and sexual orientation and race, ethnicity and racism.

Reporting on sex and gender	N/A
Reporting on race, ethnicity, or other socially relevant groupings	N/A
Population characteristics	N/A
Recruitment	N/A
Ethics oversight	N/A

Note that full information on the approval of the study protocol must also be provided in the manuscript.

Field-specific reporting

Please select the one below that is the best fit for your research. If you are not sure, read the appropriate sections before making your selection.

☒ Life sciences ☐ Behavioural & social sciences ☐ Ecological, evolutionary & environmental sciences

For a reference copy of the document with all sections, see nature.com/documents/nr-reporting-summary-flat.pdf

Life sciences study design

All studies must disclose on these points even when the disclosure is negative.

Sample size	Sample size of at least 3 independent biological replicates (unless otherwise indicated) was sufficient to establish statistical significance.
Data exclusions	Analysis of high throughput sequencing data excluded sequence reads with ambiguous nucleotides (e.g. N, S, W, R, K). Reads were discarded as described in the relevant methods section (UMIs represented below 2% oversampling or individual strands below 20% of the sum of reads for a specific UMI).<br><br>One biological replicate from Fig. 3E (PolX-/- +1x WT Pol lambda, left of dashed line) failed to amplify for any markers. We accordingly excluded data from this sample and only included the other 2 replicates. The exact same experimental conditions were replicated in three biological replicates for another experiment with an independent cell preparation on a different day (Fig. 3E, 3rd sample right of dashed line) and yielded comparable results.
Replication	Details of biological and/or technical replicates are included in the corresponding figure legends and methods sections.
Randomization	Our study is not subject to randomization since it does not involve allocation into experimental groups. Therefore, randomization is not relevant for the experiments performed.
Blinding	Quantifiable data was obtained directly from instruments, and all samples were analyzed/transformed via identical methods. Therefore, blinding was not necessary.

Reporting for specific materials, systems and methods

We require information from authors about some types of materials, experimental systems and methods used in many studies. Here, indicate whether each material, system or method listed is relevant to your study. If you are not sure if a list item applies to your research, read the appropriate section before selecting a response.

Materials & experimental systems

n/a	Involved in the study
☐	☒ Antibodies
☐	☒ Eukaryotic cell lines
☒	☐ Palaeontology and archaeology
☒	☐ Animals and other organisms
☒	☐ Clinical data
☒	☐ Dual use research of concern
☒	☐ Plants

Methods

n/a	Involved in the study
☒	☐ ChIP-seq
☒	☐ Flow cytometry
☒	☐ MRI-based neuroimaging

Antibodies

Antibodies used

PRIMARY ANTIBODIES:
Rabbit anti-Ku70 – Cell Signaling Technologies D10A7 (1:1000)
Mouse anti-pan Actin – Novus Biologicals nb600-535 (1:5000)
Rabbit anti-DNA Polymerase β – Abcam ab26343 (1:1000)

SECONDARY ANTIBODIES:
IRDye® 680LT Goat anti-Mouse IgG: LI-COR 926-68020 (1:10000)
IRDye® 800CW Goat anti-Rabbit IgG: LI-COR 926-32211 (1:10000)

Validation

Ku70 antibody: Rabbit monoclonal antibody is produced by immunizing animals with a synthetic peptide corresponding to residues surrounding Val294 of mouse Ku70 protein. Species reactivity: human, mouse, rat, monkey. Validation: Western blot analysis of extracts from various cell lines available on manufacturer's website.

Actin antibody: Mouse monoclonal antibody which reacts with all six known vertebrate isoforms of actin (approximately 42kDa). It also reacts with two cytoplasmic actins (beta, gamma) which are highly homologous to one another but which differ from the muscle actins at about 25 amino acid residues. Species reactivity: human, mouse, rat, porcine, bovine, canine, chicken, protozoa, rabbit. Validation: Western blot analysis from various mouse and human cells lines available on manufacturer's website.

DNA Polymerase β antibody: Rabbit polyclonal antibody. The exact immunogen used to generate this antibody is proprietary information (Abcam). Species reactivity: human, mouse, rat. Validation: Western blot analysis from various mouse, rat, and human cells lines available on manufacturer's website.

Eukaryotic cell lines

Policy information about cell lines and Sex and Gender in Research

Cell line source(s)

Mouse embryo fibroblasts (MEFs) were derived from E14.5d mouse embryos and SV40 T-antigen transformed - a gift from Dr. Luis Blanco
Polq-/- MEFs were derived from C57BL/6 mice - a gift from Dr. Richard Wood
HCT116 were a gift from Dr. Eric Hendrickson

Authentication

All previously unpublished cell lines (Ku-/- and PolX-/-) were validated with genotyping (sequencing of relevant alleles) and western blot analysis with comparison to the parental cell line.

Mycoplasma contamination

Spent media from cell cultures was routinely confirmed negative for mycoplasma contamination (less than 10 genomes/mL) using an rDNA amplicon.

Commonly misidentified lines
(See ICLAC register)

No commonly misidentified cell lines were used in this study.

Plants

Seed stocks	N/A
Novel plant genotypes	N/A
Authentication	N/A
